# Efficacy and Safety of Olezarsen in Patients With Hypertriglyceridemia: A Meta‐Analysis of Randomized Controlled Trials

**DOI:** 10.1155/cdr/9984822

**Published:** 2026-01-14

**Authors:** Kainat Feroz, Amna Parvez, Noor Ul Huda Ramzan, Tehreem Asghar, Ameer Haider Cheema, Ayesha Parvaiz Malik, Tehreem Fatima, Javed Iqbal, Brijesh Sathian

**Affiliations:** ^1^ Department of Medicine, King Edward Medical University, Lahore, Pakistan, kemu.edu.pk; ^2^ Department of Internal Medicine, Dow Medical College, Karachi, Pakistan, duhs.edu.pk; ^3^ Department of Internal Medicine, University of Texas at Southwestern, Dallas, Texas, USA; ^4^ Department of Internal Medicine, Akhtar Saeed Medical and Dental College, Lahore, Pakistan, amdc.edu.pk; ^5^ Department of Internal Medicine, Jinnah Sindh Medical University, Karachi, Pakistan, jsmu.edu.pk; ^6^ University of Malaya, Kuala Lumpur, Malaysia, um.edu.my; ^7^ Nursing Department, Hamad Medical Corporation Doha, Doha, Qatar; ^8^ Geriatrics and Long-Term Care Department, Rumailah Hospital, Hamad Medical Corporation, Doha, Qatar, hamad.qa

**Keywords:** apoC-III inhibitor, efficacy, hypertriglyceridemia, olezarsen, safety

## Abstract

**Aims:**

Novel drugs that target apoC‐III in lipoprotein metabolism to reduce plasma triglycerides are currently under development. Olezarsen, a drug similar to its predecessor, volanesorsen, is in Phase 3 trials. A meta‐analysis of RCTs was done to study its effect on hypertriglyceridemia.

**Material and Methods:**

Screening was done on PubMed, Embase, Scopus, and the Cochrane Library from inception to August 2024. We used Review Manager (Version 5.4) for statistical analysis. Subgroups of olezarsen at doses of 10, 50, and 80 mg were made. Continuous outcomes were reported as mean differences with 95% CIs. Adverse events were reported using risk ratios and 95% CIs. The risk of bias was analyzed using the Cochrane risk of bias tool.

**Results:**

Four RCTs with 374 participants, 278 in intervention and 96 placebo controls, were included. At all doses of olezarsen, a significant reduction in fasting triglyceride levels (MD: 45.69; 95% CI: 35.84, 55.54; *p* < 0.00001) was seen. This was most pronounced at 80 mg dose (MD: 51.98 95% CI: 43.06, 60.90; *p* < 0.00001). A reduction in apoC‐III (MD: 58.77; 95% CI: 35.94, 81.61; *p* < 0.00001) and an increase in HDL (MD: 0.21; 95% CI: 0.04, 0.37; *p* = 0.01) were also observed. No significant effect was observed on LDL levels (MD: −0.13; 95% CI: −0.40, 0.14; *p* = 0.34). Overall, no significant adverse effects were seen compared to placebo (RR: 1.42; 95% CI: 0.66, 3.05; *p* = 0.37).

**Conclusion:**

Olezarsen showed remarkable efficacy in lowering triglycerides, with a dose‐dependent effect, while significantly increasing HDL levels and reducing apoC‐III. Its safety profile is commendable. Although it performed well compared to volanesorsen, inconsistencies in LDL reduction and heterogeneity in some groups warrant further large‐scale RCTs to better assess its safety and efficacy. Clinical decisions should be tailored to individual patient profiles, as hypertriglyceridemia management may vary on a case‐to‐case basis.

## 1. Introduction

According to the American Heart Association, hypertriglyceridemia (HTG) has been defined as a serum triglyceride level of ≥ 150 mg/dL. Elevation of plasma triglycerides to a moderate level (175–499 mg/dL) has been linked to many life‐threatening conditions, such as atherosclerotic cardiovascular events, hypertension, and Type 2 diabetes mellitus. Severe elevations (≥ 500 mg/dL) have been associated with acute pancreatitis [1]. Elevated triglyceride levels reflect dysregulated lipoprotein metabolism, either from a primary genetic disorder like familial chylomicronemia syndrome (FCS) or from secondary factors such as a high cholesterol diet, obesity, smoking, alcohol intake, and poorly controlled diabetes [2].

Current treatment guidelines recommend reducing triglyceride levels to below 500 mg/dL to prevent serious complications [1]. Dietary changes, along with some available drug therapies, have proven to be successful. However, in most conditions, they are insufficient in reducing triglycerides to the desired level, particularly in cases of primary HTG [3].

Clinically, for reducing triglyceride‐rich lipoprotein levels (TRLs) and concurrent risk of cardiovascular complications, statins remain the first line of treatment, as well as other drugs like fibrates, niacin, and icosapent ethyl [3]. The majority of these traditional pharmacological agents, except fibrates, have limited success in meaningfully reducing triglyceride levels and successfully preventing pancreatic complications [3]. To address these limitations, novel therapeutic agents are in clinical development that could potentially reduce triglycerides to a degree not previously seen.

These advancements have been possible through a deeper understanding of lipoprotein metabolism and targeted interventions. Apolipoprotein C‐III (apoC‐III), a glycoprotein encoded by the APOC3 gene, is synthesized primarily in the liver and, to a lesser extent, in the small intestine. It increases plasma triglyceride levels through several mechanisms, including inhibiting lipoprotein lipase and reducing hepatic clearance of TRLs. Loss‐of‐function variants in the APOC3 gene have previously been associated with lower triglyceride levels and reduced cardiovascular risk [4]. Volanesorsen, an unconjugated antisense oligonucleotide targeting APOC3 messenger RNA (mRNA), was the first major breakthrough of its kind, showing significant triglyceride level reduction both in healthy individuals and in patients with primary HTG [5, 6]. A meta‐analysis showed that volanesorsen is associated with a reduced risk of acute pancreatitis [6].

Building on these promising results, a novel drug with a similar mechanism, olezarsen, is currently being evaluated in Phase 3 trials and showing great potential. Olezarsen is an investigational N‐acetylgalactosamine–conjugated antisense oligonucleotide targeting APOC3 mRNA. In a recent Phase 2b, randomized, controlled trial of patients with predominantly moderate HTG at elevated cardiovascular risk, olezarsen significantly reduced triglycerides, Apolipoprotein B, and non‐HDL cholesterol levels with no significant safety concerns [7].

A systematic review and meta‐analysis of clinical trials of olezarsen were performed to understand the efficacy and safety of this drug in reducing triglyceride levels in patients with HTG and to establish its effect, if any, on plasma levels of HDL, LDL, and apoC‐III.

## 2. Materials and Methods

Our meta‐analysis was conducted according to the guidance presented in the *Cochrane Handbook for Systematic Reviews of Interventions* and reported according to the Preferred Reporting Items for Systematic Reviews and Meta‐Analysis (PRISMA) statement [8, 9]. Our protocol is registered with PROSPERO (The International Prospective Register of Systematic Reviews CRD42024591396).

### 2.1. Information Sources and Search Strategy

The articles were retrieved by searching PubMed, Embase, Scopus, ScienceDirect, and the Cochrane Library from inception to August 2024. Furthermore, the reference lists were screened to retrieve more articles. Our search strategy incorporated a combination of keywords and MeSH terms such as olezarsen, GalNAc‐ASO, Apolipoprotein C inhibitor, and HTG.

### 2.2. Eligibility Criteria

The inclusion criteria were as follows: (1) patient population, adults ≥ 18 years of age with HTG (fasting TG ≥ 150 mg/dL); (2) intervention, olezarsen therapy; (3) comparison, placebo; (4) outcome, reporting at least one outcome of interest; and (5) study design, randomized controlled trials (RCTs) only.

The exclusion criteria were as follows: (1) all studies other than RCTs, like review articles, and (2) triglyceride levels below 150 mg/dL.

### 2.3. Selection Process

**Figure 1:**
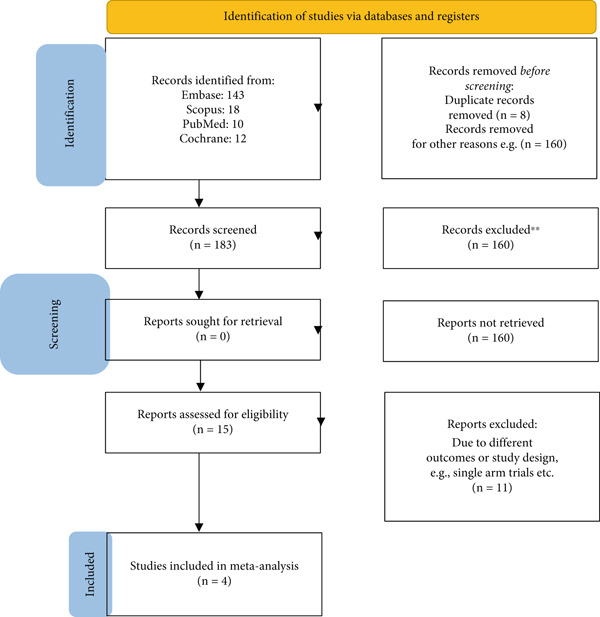
PRISMA flowchart.

We imported all the studies obtained through our online search into Rayyan [10] and removed duplicate articles. Two authors independently performed title and abstract screening and excluded irrelevant articles. We then performed full‐text screening on the remaining studies and finalized the studies conforming to our eligibility criteria. A third author settled any disagreements over the selection of studies. The selection process is presented in the form of a PRISMA flowchart (Figure 1).

### 2.4. Data Collection Process and Data Items

Two independent authors extracted data from the finalized articles into a structured Excel sheet, and any discrepancy was resolved by consulting with the corresponding author. The extracted data included baseline demographic characteristics such as age and sex, study characteristics such as author names, study duration, follow‐up period, sample size, and intervention and comparator details such as type of drug, dose frequency, duration, and outcomes.

### 2.5. Outcome Measures

The primary outcome of interest was the reduction in triglyceride levels from the baseline. The secondary outcomes of interest were a reduction in apoC‐III levels, a reduction in LDL, and an increase in HDL. Various adverse events were also analyzed.

### 2.6. Risk of Bias Assessment

The bias risks of our included trials were analyzed using the Cochrane risk of bias tool for randomized trials (RoB 2) [11], which assesses bias in the following five domains: (1) bias arising from the randomization process, (2) bias caused by deviations from intended interventions, (3) bias caused by missing outcome data, (4) bias in the measurement of the outcome, and (5) bias in the selection of the reported result. Two authors independently rated the risk of bias for each included study as low, high, or some concerns. A third reviewer resolved any disagreement between them.

### 2.7. Data Synthesis

We used Review Manager (RevMan, Version 5.4; The Cochrane Collaboration, Copenhagen, Denmark) for statistical analysis [12]. We reported continuous outcomes as mean difference (MD) with 95% CIs. Subgroups were made according to the dosage of olezarsen administered: 10, 50, and 80 mg for all outcomes, respectively. The adverse events were analyzed using risk ratio and 95% CI. We used a random effects model to perform meta‐analyses. For each synthesis, we calculated the chi^2^ test and *I* ^2^ statistic to detect and quantify the presence of heterogeneity, respectively. We interpreted *I* ^2^ values according to the *Cochrane Handbook for Systematic Reviews of Interventions* [8]. *p* < 0.10 was considered statistically significant for the chi^2^ test. We planned to assess publication bias using funnel plots if the number of studies was more than 10.

## 3. Results

### 3.1. Study Selection and Characteristics

A total of 183 studies were retrieved through our database search. After screening, a total of four RCTs [7, 13–15] were included in our review. The detailed screening process is given in Figure 1.

A total of 374 participants, 278 receiving olezarsen therapy and 96 placebo‐treated controls, were included in the final analysis. All trials administered a single dose of olezarsen, 10–80 mg, once every 4 weeks with a treatment period of 3 months to 1 year. Trials had participants aged 18–65 years from the United States and Canada. The detailed study characteristics are given in Table 1.

**Table 1:** Characteristics of included RCTs.

**Study name**	**Study design**	**Country**	**Study period**	**Sample size**	**Mean age**	**Male/female ratio**	**Baseline TAG**	**Dose (mg)**	**Dose frequency**	**Treatment period**	**Follow-up**	**Comorbid diseases**
Bergmark et al. 2024 [7]	RCT (Phase 2b)	United States and Canada	June through September 2022	154 total: 58 (50 mg), 57 (80 mg), 39 (placebo)	62	89 M/65 F	241.5 mg/dL median	50 or 80 mg	Once a month	12 months	3.24 months (13 weeks)	Pancreatitis, diabetes mellitus, chronic kidney disease, atherosclerotic cardiovascular disease
Tardif et al. 2022 [14]	RCT (Phase 2)	United States and Canada	6 months	114 total: 22 (10 mg), 22 (50 mg), 23 (15 mg), 23 (10 mg)	65.3 ± 8.10	86 M/28 F	262 (222–329) mg/dL median IQR	10 or 15 or 50 mg	Every 4 weeks (10 or 50 mg) or every 2 weeks (15 mg) or every week (10 mg)	6.68 months (placebo), 7.73 months (olezarsen)	6.25–6.75 months (25–27 weeks)	Hypertension, Type 2 diabetes, coronary artery disease, acute myocardial infarction
Stroes et al. April 2024 [15]	RCT (Phase 3)	United States, Canada, Europe (11 countries)	November 2020 through October 2023	66 total: 21 (50 mg), 22 (80 mg), 23 (placebo)	18+	28 M/38 F	2630 ± 1315 mg/dL mean SD	50 or 80 mg	Once every 4 weeks	12.25 months (49 weeks)	6 months; 12 months	Acute pancreatitis, Type 1 or 2 diabetes mellitus, hypertension, thrombocytopenia
Alexander et al. 2019 [13]	RCT (Phase 1/2a)	United States	3 months	40 total: 6 (10 mg), 6 (30 mg), 6 (60 mg), 6 (90 mg), 6 (120 mg)	18–65	23 M/17 F	≥ 90 or ≥ 200 mg/dL	Single dose 10, 30, 60, 90, and 120 mg and multiple dose 15 and 30 or 60 mg	Single dose 10, 30, 60, 90, and 120 mg and multiple dose cohorts 15 and 30 mg weekly or 60 mg every 4 weeks	1.5–3 months	7–14 days	NR

Two out of four trials had patients with the highest risk of cardiovascular complications with triglyceride levels greater than 200 mg/dL. They were all given olezarsen therapy along with standard‐of‐care preventative therapy for cardiovascular risks [7, 14]. One trial evaluated asymptomatic patients with elevated triglyceride levels who only received olezarsen as monotherapy [13]. Another study evaluated a reduction in TGL levels in patients with genetically identified familial chylomicronemia [16]. Furthermore, three out of four clinical trials had participants with diabetes and cardiovascular complications as comorbidities [7, 14, 15]. Two studies also included participants with a history of acute pancreatitis, a recognized adverse effect of high triglyceride levels [7, 15].

### 3.2. Risk of Bias in Included Studies

**Figure 2:**
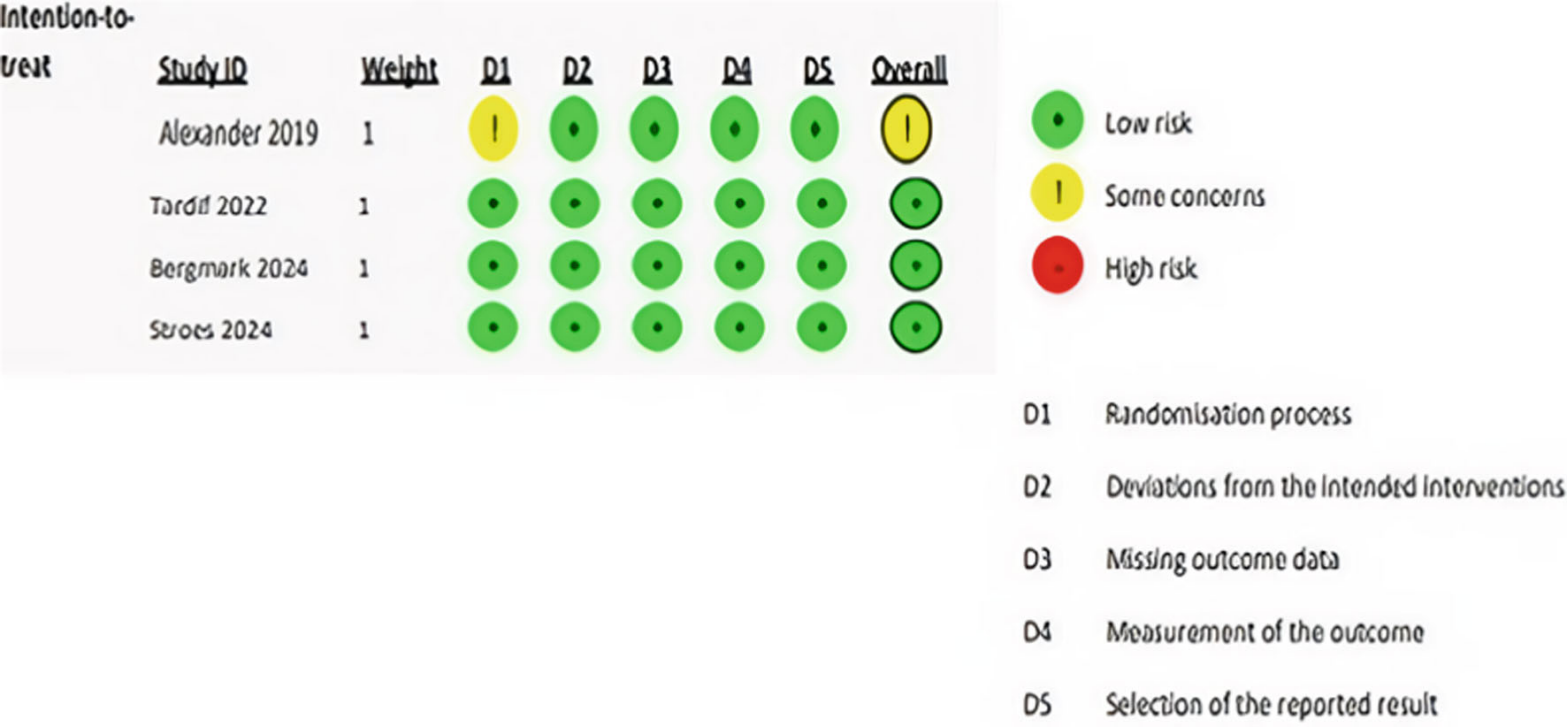
Risk of bias in included studies.

Overall, three studies (75%) were judged to be at low risk of bias, while one study (25%) was found to be at some risk of bias, mostly in the randomization process. The most common flaw was a lack of data about allocation concealment and the lack of a trial registry record containing an analysis plan. The risk of bias table is presented in Figure 2.

### 3.3. Results of Syntheses

#### 3.3.1. Comparison: Olezarsen Versus Placebo

##### 3.3.1.1. Primary Outcome: Reduction in Triglyceride Levels

**Figure 3:**
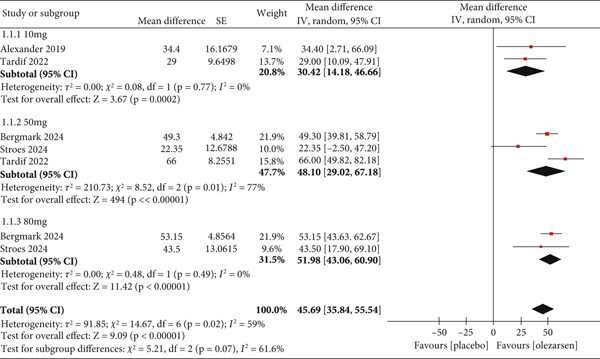
Forest plot displaying the mean difference and 95% confidence intervals for the effect of olezarsen on plasma levels of triglyceride at 80, 50, and 10 mg dosage.

Compared with a placebo, a single dose of olezarsen every 4 weeks showed a significant reduction in fasting triglyceride levels (MD: 45.69; 95% CI: 35.84, 55.54; *p* < 0.00001; *I* ^2^ = 59*%*; Figure 3). However, a more substantial decrease in triglyceride level was observed with higher doses of 80 mg (MD: 51.98; 95% CI: 43.06, 60.90; *p* < 0.00001; *I* ^2^ = 0) and 50 mg (MD: 48.10; 95% CI: 29.02, 67.18; *p* < 0.00001; *I* ^2^ = 77*%*) as compared to a low dose of 10 mg (MD: 30.42; 95% CI: 14.18, 46.66; *p* = 0.0002; *I* ^2^ = 0), respectively. Conclusively, high‐dose olezarsen therapy was associated with a robust reduction in triglyceride levels.

##### 3.3.1.2. Secondary Outcomes: Reduction in apoC‐III Levels

**Figure 4:**
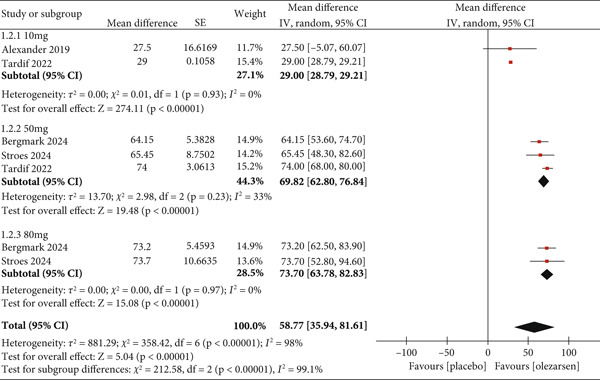
Forest plot displaying the mean difference and 95% confidence intervals for the effect of olezarsen on plasma levels of apoC‐III at 80, 50, and 10 mg dosage.

A reduction in apoC‐III levels was observed at all doses of olezarsen (MD: 58.77; 95% CI: 35.94, 81.61; *p* < 0.00001; *I* ^2^ = 98*%*; Figure 4). Similar to its effect on triglycerides, 80 mg (MD: 73.3; 95% CI: 63.78, 82.83; *p* < 0.00001; *I* ^2^ = 0) and 50 mg (MD: 69.82; 95% CI: 62.80, 76.84; *p* < 0.00001; *I* ^2^ = 33*%*) showed a greater reducing effect than a low dose of 10 mg (MD: 29.00; 95% CI: 28.79, 29.21; *p* < 0.00001; *I* ^2^ = 0).

#### 3.3.2. Increase in HDL

**Figure 5:**
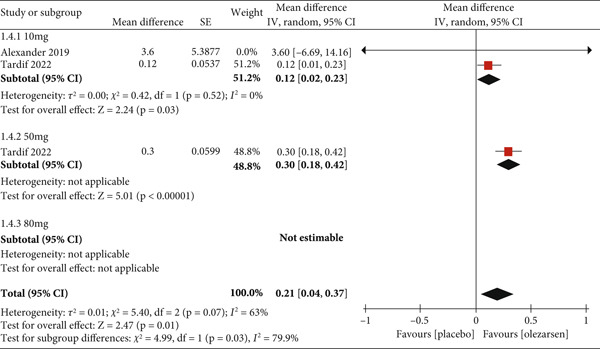
Forest plot displaying the mean difference and 95% confidence intervals for the effect of olezarsen on plasma levels of HDL at 80, 50, and 10 mg dosage.

Olezarsen was associated with an increase in HDL levels at 50 mg (MD: 0.30; 95% CI: 0.18, 0.42; *p* < 0.00001) and 10 mg (MD: 0.12; 95% CI: 0.02, 0.23; *p* = 0.03; *I* ^2^ = 0). The effect of olezarsen at the high dose of 80 mg on HDL levels could not be evaluated. The overall effect of olezarsen on HDL levels was statistically significant compared to placebo (MD: 0.21; 95% CI: 0.04, 0.37; *p* = 0.01; *I* ^2^ = 63*%*; Figure 5).

#### 3.3.3. Reduction in LDL

**Figure 6:**
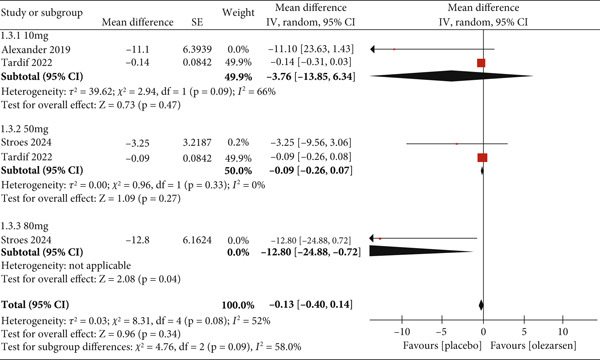
Forest plot displaying the mean difference and 95% confidence intervals for the effect of olezarsen on plasma levels of LDL at 80, 50, and 10 mg dosage.

Olezarsen showed no significant effect on LDL levels compared to placebo (MD: −0.13; 95% CI: −0.40, 0.14; *p* = 0.34; *I* ^2^ = 52*%*; Figure 6). However, 80 mg showed a statistically significant increase (MD: −12.80; 95% CI: −24.88, −0.72; *p* = 0.04), while no significant result was observed in those receiving a dose of 50 mg (MD: −0.09; 95% CI: −0.26, 0.07; *p* = 0.27; *I* ^2^ = 0) and 10 mg (MD: −3.76; 95% CI: −13.85, 6.34; *p* = 0.47; *I* ^2^ = 66*%*).

### 3.4. Safety Analysis

**Figure 7:**
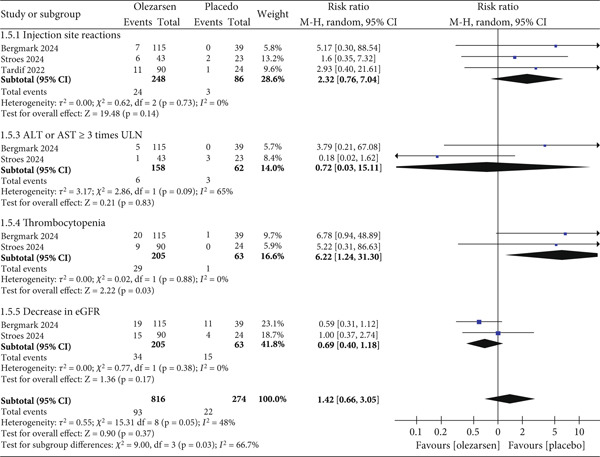
Forest plot displaying the risk ratio and 95% confidence intervals for common side effects of olezarsen versus placebo.

In comparison of olezarsen with placebo, the most commonly reported adverse events in all clinical trials were injection site reactions (RR: 2.32; 95% CI: 0.76, 7.04; *p* = 0.14; *I* ^2^ = 0*%*), ALT and AST ≥ 3 times the ULN (upper limit of normal) (RR: 0.72; 95% CI: 0.03, 15.11; *p* = 0.83; *I* ^2^ = 65*%*), a platelet count of less than 140,000/*μ*L (RR: 6.22; 95% CI: 1.24, 31.30; *p* = 0.03; *I* ^2^ = 0*%*), and a decrease in eGFR ≥ 25% (RR: 0.69; 95% CI: 0.04, 1.18; *p* = 0.17; *I* ^2^ = 0*%*). However, they were all nonsignificant except for the decrease in platelet count. Overall, the adverse effects of olezarsen compared to placebo were not significant (RR: 1.42; 95% CI: 0.66, 3.05; *p* = 0.37; *I* ^2^ = 48*%*; Figure 7) [7, 13–15].

Other side effects also included headache, adverse events leading to treatment discontinuation, urine protein/creatinine ratio > 500 mg/g, elevation in serum creatinine, pancreatitis, and flu‐like illness; however, these were all low in number and not statistically significant. Any severe reaction or mortality reported in trials was ruled to not be drug‐related. No significant bleeding event as a result of thrombocytopenia was reported. No severe renal or hepatic abnormalities were reported.

## 4. Discussion

Recent experimentation with olezarsen is ongoing to target apoC‐III production to potentially treat HTG. To our knowledge, this is a novel meta‐analysis that discusses the various outcomes of olezarsen, including all the available studies related to this.

Compared with placebo, the 10, 50, and 80 doses of olezarsen significantly reduced triglyceride levels. HDL levels were also significantly increased among the 10 and 50 mg groups but were not estimable for the 80 mg group. The levels of apoC‐III showed a significant reduction across all three groups, which is beneficial for the lipid profile. There was no significant effect of olezarsen on LDL levels.

Regarding triglyceride levels, there was an overall reduction of 45.69 (MD). The results in all groups significantly favored olezarsen, with a reduction of 30.42 (MD) in the 10 mg group from the baseline compared to the placebo. The 50 mg group showed a reduction of 48.10 (MD), and the 80 mg group reported a 51.98 (MD) reduction. Therefore, our analysis shows that olezarsen has a dose‐dependent effect, with the 80 mg dose giving the best results. One study mentioned that 6 months of 50 or 80 mg olezarsen led to reduced levels of triglycerides by nearly 50% in individuals with HTG and increased cardiovascular risks [7]. Previously, a nonconjugated form of apoC‐III inhibitor known as “volanesorsen” has been studied for HTG. One study showed that volanesorsen led to a statistically significant reduction in triglyceride levels, with an MD of 73.9% after 3 months of treatment compared to the placebo group [16]. Another study showed an MD of 78.85% between the volanesorsen and placebo groups. However, these results were statistically nonsignificant [16]. Fogacci et al. reported a significant MD of 67.90% between the volanesorsen and placebo groups [17]. Triglyceride levels were reportedly reduced from > 5000 mg/dL (56 mmol/L) to 350–500 mg/dL (4–5.6 mmol/L) in one patient [17]. A clinical trial (COMPASS study) demonstrated that triglyceride levels were significantly reduced by 71.2% in the volanesorsen group compared to the placebo group [5]. This reduction in triglyceride levels can be explained by the mechanism of action of olezarsen and volanesorsen, which comprises apoC‐III inhibition. apoC‐III is mainly formed in the intestine and liver and has a high affinity for the surface of lipoprotein particles [18, 19]. It hinders the body′s ability to clear triglycerides. Thus, inhibition of this protein allows the body to rid itself of the triglycerides [7]. Various human genetic studies have shown that individuals with apoC‐III loss‐of‐function mutation have a reduced level of triglycerides, lower non‐HDL‐C, and higher HDL‐C levels [18].

The levels of apoC‐III showed a significant overall reduction of 58.77 (MD) when compared to the placebo: 10 mg at 29.00 (MD), 50 mg at 69.82 (MD), and 80 mg at 73.30 (MD). Calcaterra et al. reported a significant reduction of 80.0% in apoC‐III levels in the volanesorsen group [16]. A significant reduction of 74.83% was reported in apoC‐III levels in another study done in patients taking volanesorsen [17]. A nonsignificant reduction of 80.08% was shown in another study conducted on volanesorsen [16]. The reduction of apoC‐III level is vital in reducing cardiovascular disease risk. These particles contribute to various steps in inducing atherosclerotic changes, such as interaction with the LDL particles, myocyte adhesion, smooth muscle proliferation, and increasing oxidative stress [20]. An experiment linked apoC‐III level reduction to a decreased vascular adhesiveness by suppressing VCAM‐1 [20]. Thus, reduction in apoC‐III levels is an essential therapeutic target for drugs to treat HTG.

When comparing the HDL levels between the olezarsen and placebo groups, there was an overall increase of 0.21 (MD). The patients in the 10 mg group showed a statistically significant increase of 0.12 (MD). The patients taking the 50 mg dose demonstrated a significant rise of 0.30 (MD). The individuals in the 80 mg dose were not estimable. Cheng et al. reported a significant increase in the HDL‐C by 46.01% in the volanesorsen group compared to the placebo group [16]. One study showed a significant increase in HDL‐C levels by 40.06% [17]. Another study reported increased HDL levels by 45.92% [16]. These results were statistically significant across all the studies mentioned above. apoC‐III is involved in HDL metabolism as well. It is mainly transferred from very low‐density lipoprotein (VLDL) to HDL, forming a large‐sized HDL3 particle in the bloodstream, further undergoing esterification of its surface cholesterol particles [21]. The HDL particles exert a protective effect and have a proven inverse association with high‐risk cardiovascular events [22, 23]. Thus, owing to the beneficial effects of HDL, multiple drugs have been developed over the past few decades to increase its levels in high‐risk individuals [24–27]. The increase in HDL levels by olezarsen is an additional advantage alongside its triglyceride‐lowering effects.

With regard to LDL, there was no significant effect. The 10 mg dose of olezarsen showed a nonsignificant increase of 3.76 (MD), and the 50 mg dose demonstrated a nonsignificant increase of 0.09 (MD) compared to the placebo. The patients taking an 80 mg dose included only one study, with a statistically significant increase of 12.80 (MD) compared to the placebo. A previous study with volanesorsen demonstrated an increase in LDL levels with an MD of 99.59% compared with the placebo group, but these results were nonsignificant [16]. Fogacci et al. reported a nonsignificant impact on LDL levels with an MD of 47.01% [17]. At the same time, LDL levels were reportedly increased in one study by 68.6% in the volanesorsen group compared to the placebo group [16]. The aforementioned results highlight a nonsatisfactory reduction in LDL levels through the inhibition of apoC‐III by olezarsen or its nonconjugated counterpart, volanesorsen. This could be explained by the inability to assess the LDL particle sizes, which might identify the large‐sized de novo LDL and the smaller LDL particles with a higher atherogenic adverse effect [28–30]. Another phenomenon that might cause increased LDL particles is the removal of triglyceride‐rich lipoproteins through a lipoprotein lipase–independent pathway. The liver takes up the triglyceride‐rich lipoproteins inhibition from the bloodstream [31, 32]. The inhibition of apoC‐III further enhances the liver′s ability to clear triglyceride‐rich particles, eventually leading to a rise in LDL particles [33].

The most significant side effects reported in clinical trials of volanesorsen were episodes of severe thrombocytopenia of up to < 25,000 platelets per microliter and associated thrombocytopenic events leading to discontinuation of drug [34]. In comparison, to date, no trial of olezarsen reported a decrease in platelet count less than 50,000 per microliter and concurrent episodes of bleeding. Reports of injection site reactions are also mild and significantly lower in trials conducted up until now, even with exposure of up to 12 months, representing a significant improvement over its counterpart. All reported adverse effects have minor incidence and are statistically nonsignificant, except a decrease in platelets to less than 140,000, but no thrombocytopenic adverse events due to it have been reported. Based on these safety and efficacy findings, olezarsen shows robust results in lower doses (50 mg compared to 1200 mg per month) and less frequent doses, representing an excellent improvement over volanesorsen till now. The drug was also not associated with any significant renal or hepatic abnormalities.

### 4.1. Strengths and Limitations

While assessing the results of this meta‐analysis, the limitations should be considered. The number of studies included was scarce, as some RCTs have yet to release their results. Some studies did not include all the mentioned dosages of the drug in question. Although the inclusion criteria were met, there was a slight variability in the patient characteristics across the included studies. The duration of drug administration was different and should be kept in mind. The data provided regarding the effects of olezarsen on Apolipoprotein B and VLDL levels was not sufficient to be analyzed. Furthermore, the overall heterogeneity was considerable in the LDL group at 52%, the HDL group at 63%, triglycerides at 59%, apoC‐III at 98%, and adverse effects at 48%. This might have an unfavorable effect on the outcomes and should be considered.

Aside from these limiting factors, this is the first meta‐analysis specifically comparing the outcomes between olezarsen and placebo. Herein, we discussed the changes in the levels of triglycerides, LDL, HDL, and apoC‐III particles across the different olezarsen dosages and assessed its safety profile. This study showed that olezarsen achieved all the abovementioned results at lower doses of 10, 50, or 80 mg, whereas volanesorsen achieved these results at 300 mg in one study [16]. Our study was conducted to provide some insight into the clinical advantages of this new drug in managing HTG. However, additional RCTs with a larger patient population must be undertaken to ensure accurate results and further explore the adverse events meticulously.

### 4.2. Implications for Clinical Practice and Research

This study sheds light on the outcomes associated with different dosages of olezarsen compared to placebo and its safety. Although there were satisfactory results for triglyceride levels, apoC‐III, and HDL levels, the same cannot be said about LDL levels. Its safety profile is praiseworthy. The heterogeneity was considerable in some groups. Thus, more RCTs need to be carried out with a sizable, well‐defined patient population for a longer duration to clarify the disagreeable LDL results and heterogeneity further. This will help justify all the outcomes more clearly and provide us with supplementary clinical evidence regarding olezarsen usage. To emphasize, clinical decisions should only be made according to the specific patient characteristics, and the management of HTG may vary on a case‐to‐case basis.

## 5. Conclusion

Olezarsen demonstrated remarkable performance in reducing triglycerides in a dose‐dependent manner, the most substantial being in the 80 mg group. Additionally, it led to significantly increased HDL levels. A distinct benefit is the significant reduction in apoC‐III, which enhances its lipid‐lowering profile. Olezarsen appears to have achieved similar results with lower and less frequent dosages than its counterpart, volanesorsen. There are also fewer adverse effects reported in comparison, and no severe thrombocytopenia (< 50,000/*μ*L) was observed in olezarsen versus volanesorsen. These outcomes constitute an essential component of the HTG treatment. Our study was conducted to provide some insight into the clinical advantages of this new drug in managing HTG. However, additional RCTs with a larger patient population must be undertaken to ensure accurate results and further explore the adverse events meticulously.

## Ethics Statement

We confirm that we have read the journal′s position on issues involved in ethical publication and affirm that this report is consistent with those guidelines.

## Disclosure

The abstract of this meta‐analysis was previously presented at the ACC 25 conference held in Chicago. All authors read and approved the final manuscript and agree to be accountable for the research presented.

## Conflicts of Interest

The authors declare no conflicts of interest.

## Author Contributions

All authors contributed to the study conception and design. **Kainat Feroz**: conceptualization, data curation, methodology, formal analysis, and supervision; **Noor Ul Huda Ramzan**: data curation, methodology, and writing original manuscript; **Amna Parvez**: data curation, methodology, writing original manuscript, introduction, and result portion; **Tehreem Asghar**: data curation, methodology, and writing original manuscript; **Ameer Haider Cheema**: writing original manuscript, discussion portion, review, and editing; **Ayesha Parvaiz Malik**: writing original manuscript, discussion portion, review, and editing; **Tehreem Fatima**: data curation, methodology, and writing original manuscript; **Javed Iqbal**: writing original manuscript, review, and supervision; **Brijesh Sathian**: mentor of the study.

## Funding

This study was funded by the Qatar National Library (10.13039/100019779).

## Data Availability

Data will be provided on reasonable request from the corresponding author.
